# Serum protein profiling reveals mechanism of activated thrombus formation in patients with stroke and atrial fibrillation

**DOI:** 10.1038/s41598-024-64750-w

**Published:** 2024-06-17

**Authors:** Sora Mun, Jae Guk Kim, Soo Joo Lee, Doojin Kim, Jiyeong Lee, Hee-Gyoo Kang

**Affiliations:** 1 Department of Biomedical Laboratory Science, College of Health Sciences, Eulji University, Seongnam, 13135 Republic of Korea; 2 Department of Neurology, Daejeon Eulji Medical Center, Eulji University School of Medicine, Daejeon, 35233 Republic of Korea; 3 Department of Hospital Business, Siotmedi Co., Ltd, Suwon, 16630 Republic of Korea; 4 Department of Biomedical Laboratory Science, College of Health Science, Eulji University, Uijeongbu, 11759 Republic of Korea

**Keywords:** Atrial fibrillation, Thrombus formation, Inflammation, Protein profiling, Stroke, Biomarkers, Diseases

## Abstract

Stroke is an acute cerebrovascular disease in which blood flow to the brain is suddenly disrupted, causing damage to nerve cells. It involves complex and diverse pathophysiological processes and the treatment strategies are also diverse. The treatment for patients with stroke and atrial fibrillation (AF) is aimed at suppressing thrombus formation and migration. However, information regarding the protein networking involved in different thrombus formation pathways in patients with AF and stroke is insufficient. We performed protein profiling of patients with ischemic stroke with and without AF to investigate the mechanisms of thrombus formation and its pathophysiological association while providing helpful information for treating and managing patients with AF. These two groups were compared to identify the protein networks related to thrombus formation in AF. We observed that patients with ischemic stroke and AF had activated inflammatory responses induced by C-reactive protein, lipopolysaccharide-binding protein, and alpha-1-acid glycoprotein 1. In contrast, thyroid hormones were increased due to a decrease in transthyretin and retinol-binding protein 4 levels. The mechanism underlying enhanced cardiac activity, vasodilation, and the resulting thrombosis pathway were confirmed in AF. These findings will play an essential role in improving the prevention and treatment of AF-related stroke.

## Introduction

A stroke is an acute cerebrovascular disease in which the sudden disruption or rupture of cerebral blood vessels damages nerve cells^1^. Strokes are generally classified into two types: ischemic, which occurs due to a decrease or blockage of cerebral blood flow, primarily by thrombus formation or embolism^2^, and hemorrhagic, which involves bleeding into the brain tissue due to the ruptured blood vessels^3^. Each type involves various pathophysiological processes with distinct causes and intricate mechanisms.

The fundamental goals of stroke treatments are minimizing brain tissue damage, preventing complications, and facilitating functional recovery^1^. However, treatment may vary due to the complex pathophysiological processes involved in the condition. Specifically, in patients with stroke and atrial fibrillation (AF), anticoagulation therapy is necessary to inhibit blood clot formation, as an irregular blood flow in the atria can lead to blood clotting^4^.

Furthermore, in patients with stroke and AF, blood clotting risk is increased, thereby increasing the risk of post-stroke complications^5^. Hence, understanding the mechanisms underlying blood clot formation due to AF in patients with stroke is crucial for stroke prevention, treatment, and management. However, the precise protein networks contributing to increased blood clot formation in patients with stroke and AF have not been conclusively identified.

Therefore, this study aimed to profile proteins of stroke patients with and without AF to identify the protein networks involved in thrombus formation. By providing crucial information for managing and treating patients with AF-related stroke, this study lays the foundation for future clinical interventions and the development of preventive strategies.

## Results

### Sample distribution

**Figure 1:**
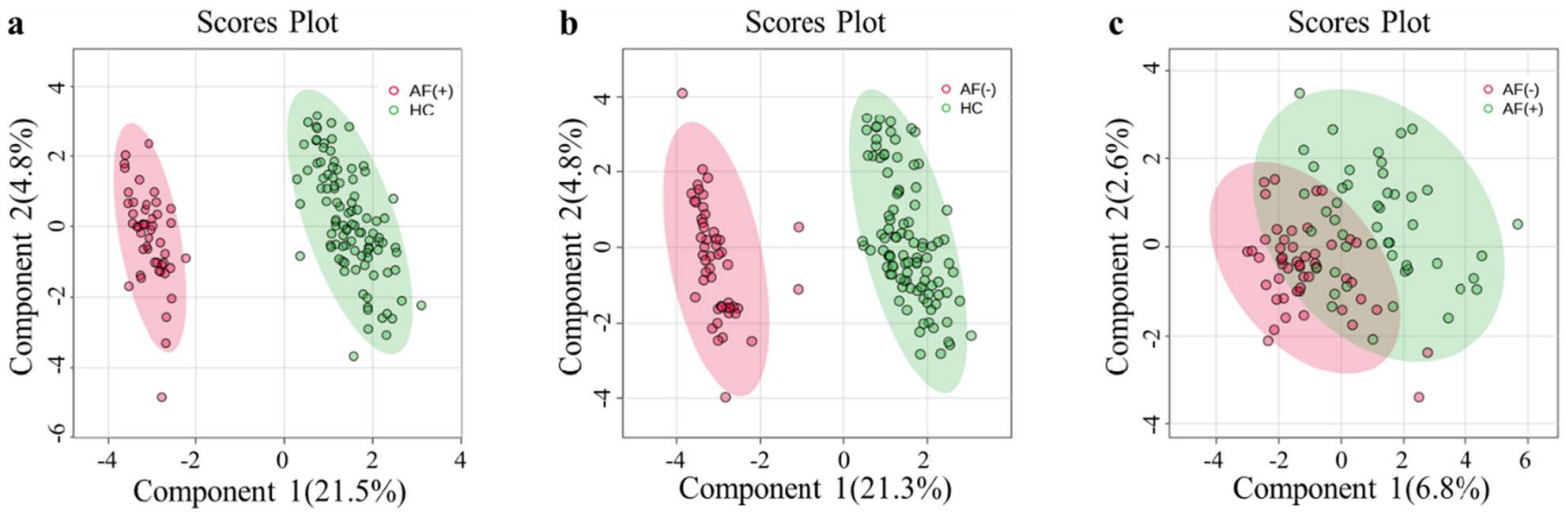
Distribution of proteins identified using LC–MS/MS in each group. (**a**) Patients with stroke and atrial fibrillation and healthy controls were distributed as components 1 (21.5%) and 2 (4.8%), respectively. (**b**) Patients with stroke without atrial fibrillation and healthy controls were distributed as components 1 (21.3%) and 2 (4.8%), respectively. (**c**) Patients with stroke and atrial fibrillation and those without atrial fibrillation were distributed as components 1 (6.8%) and 2 (2.6%), respectively. LC–MS/MS, liquid chromatography-tandem mass spectrometry.

Table 1 shows the demographic information of the samples. A sparse partial least squares discriminant analysis was conducted to compare patients with stroke and AF with those without AF. Patients with stroke and AF and healthy controls were classified as components 1 (21.5%) and 2 (4.8%), respectively (Fig. 1a). Patients with stroke without AF and healthy controls were classified as components 1 (21.3%) and 2 (4.8%), respectively (Fig. 1b). Patients with stroke and AF and those with stroke without AF were classified as component 1 (6.8%) and component 2 (2.2%), respectively (Fig. 1c). We illustrated the difference in protein levels between patients with stroke and AF and those without AF.

### Comparative analysis of patients with stroke with or without atrial fibrillation

**Figure 2:**
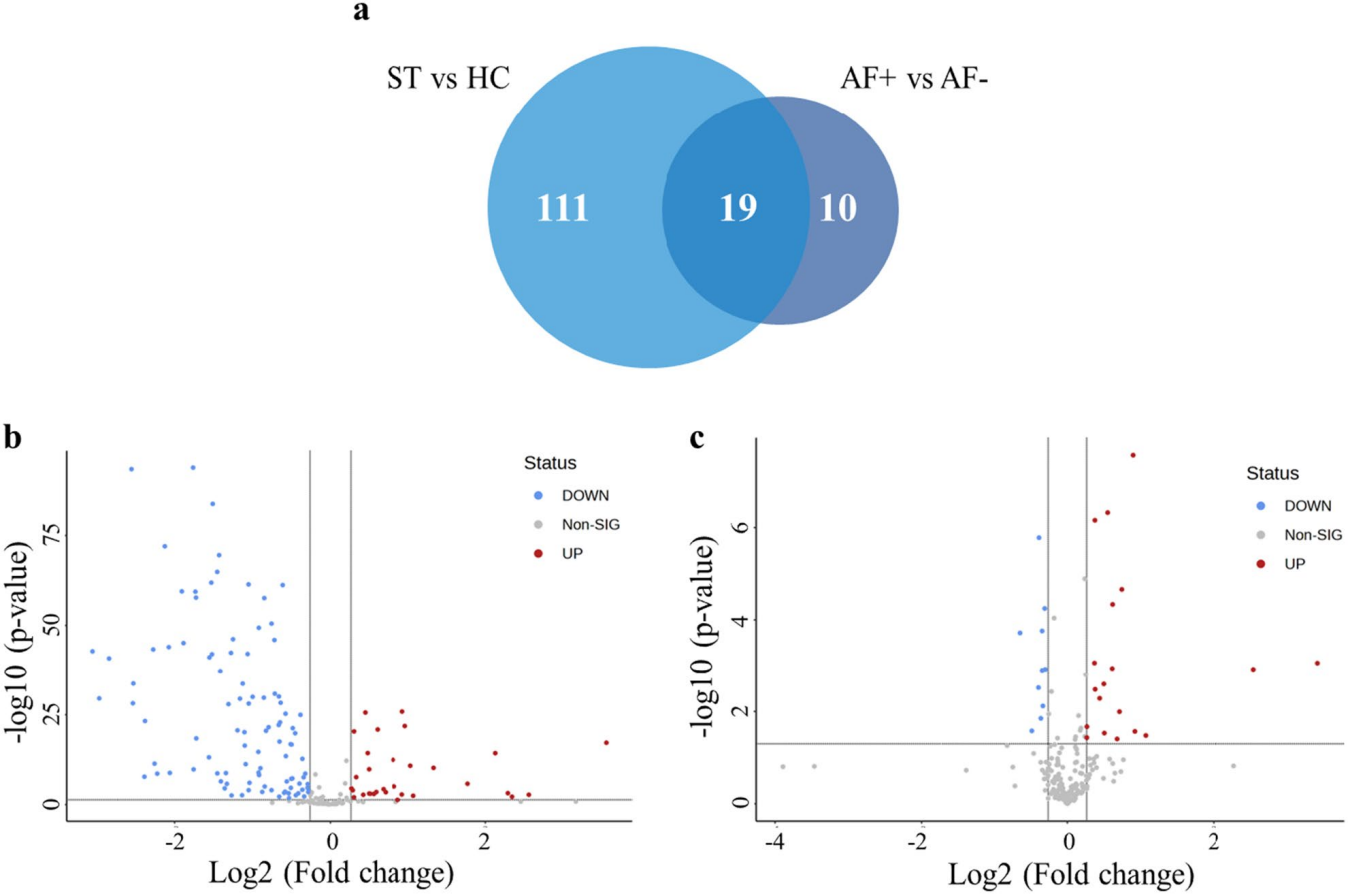
Venn diagram and volcanoplot analysis of statistically different proteins in each group. (**a**) Venn diagram of differentially expressed proteins (*p* < 0.05) in patients with stroke (ST) compared to the healthy controls (HC); and those differentially expressed in patients with stroke and atrial fibrillation (AF +) compared to patients with stroke without atrial fibrillation (AF-). (**b**–**c**) Volcano plot analysis of differentially expressed proteins (*p* < 0.05) in patients with stroke compared to the healthy controls or patients with stroke and atrial fibrillation compared to patients with stroke without atrial fibrillation.

Protein profiling of blood samples from patients with stroke either with or without AF was performed to identify the altered proteins in patients with stroke and AF and their associations with thrombus formation. We identified proteins expressed differentially in patients with stroke and AF compared to those in patients with stroke without AF. Of the 194 identified proteins, 29 were differentially expressed in patients with stroke and AF, more than a 1.2-fold change compared to those in the healthy control group (*p* < 0.05; Fig. 2). In addition, we identified differentially expressed proteins in the healthy control group compared with those in the stroke group. This study aimed to identify proteins that specifically differ in patients with AF during stroke and reveal the mechanisms through which such proteins are involved in the pathophysiology of AF. Therefore, we first determined the differentially expressed proteins following the stroke events. We identified proteins that were differentially expressed in patients with stroke compared to the healthy control group, as well as those that were differentially expressed in patients with stroke and AF compared to patients with stroke without AF. Of the 194 identified proteins, 130 were differentially expressed in patients with stroke with over a 1.2-fold change compared to those in the control group (*p* < 0.05). Finally, we selected 19 proteins that were commonly identified among those showing differential expression in stroke patients compared to healthy controls, and in patients with AF-related stroke compared to those without AF, as candidate proteins for distinguishing AF-related stroke from stroke without AF (Table 2). Of the 19 proteins selected, 11 had a pathophysiological relation to stroke and were selected based on a literature search (Table 2).

**Table 1:** List of candidate proteins markers specific for stroke patients with atrial fibrillation.

No	Accession name	Entry name	Protein name	Stroke vs. control		With AF vs. without AF	
				*p*-value	Fold change	*p*-value	Fold change
1	Q06033	ITIH3	Inter-alpha-trypsin inhibitor heavy chain H3	4.89E-08	1.86	9.84E-37	0.38
2	P02766	TTHY	Transthyretin	1.62E-06	0.77	3.02E-13	0.78
3	P02753	RET4	Retinol-binding protein 4	0.00018	0.64	1.3E-20	0.56
4	P02743	SAMP	Serum amyloid P-component	0.00117	0.81	0.0001	1.22
5	P18428	LBP	Lipopolysaccharide-binding protein	0.0014	1.53	5.7E-11	2.05
6	P0DJI8	SAA1	Serum amyloid A-1 protein	0.00151	10.74	0.00217	5.89
7	P02741	CRP	C-reactive protein	0.00188	5.86	0.00083	4.89
8	P02763	A1AG1	Alpha-1-acid glycoprotein 1	0.00366	1.30	6.08E-13	1.75
9	P02655	APOC2	Apolipoprotein C-II	0.01362	0.78	6.9E-08	0.71
10	P13591	NCAM1	Neural cell adhesion molecule 1	0.02808	1.89	3.23E-06	3.41
11	P35542	SAA4	Serum amyloid A-4 protein	0.02917	1.42	3E-42	0.35

### MRM analysis for verification of candidate biomarkers

**Figure 3:**
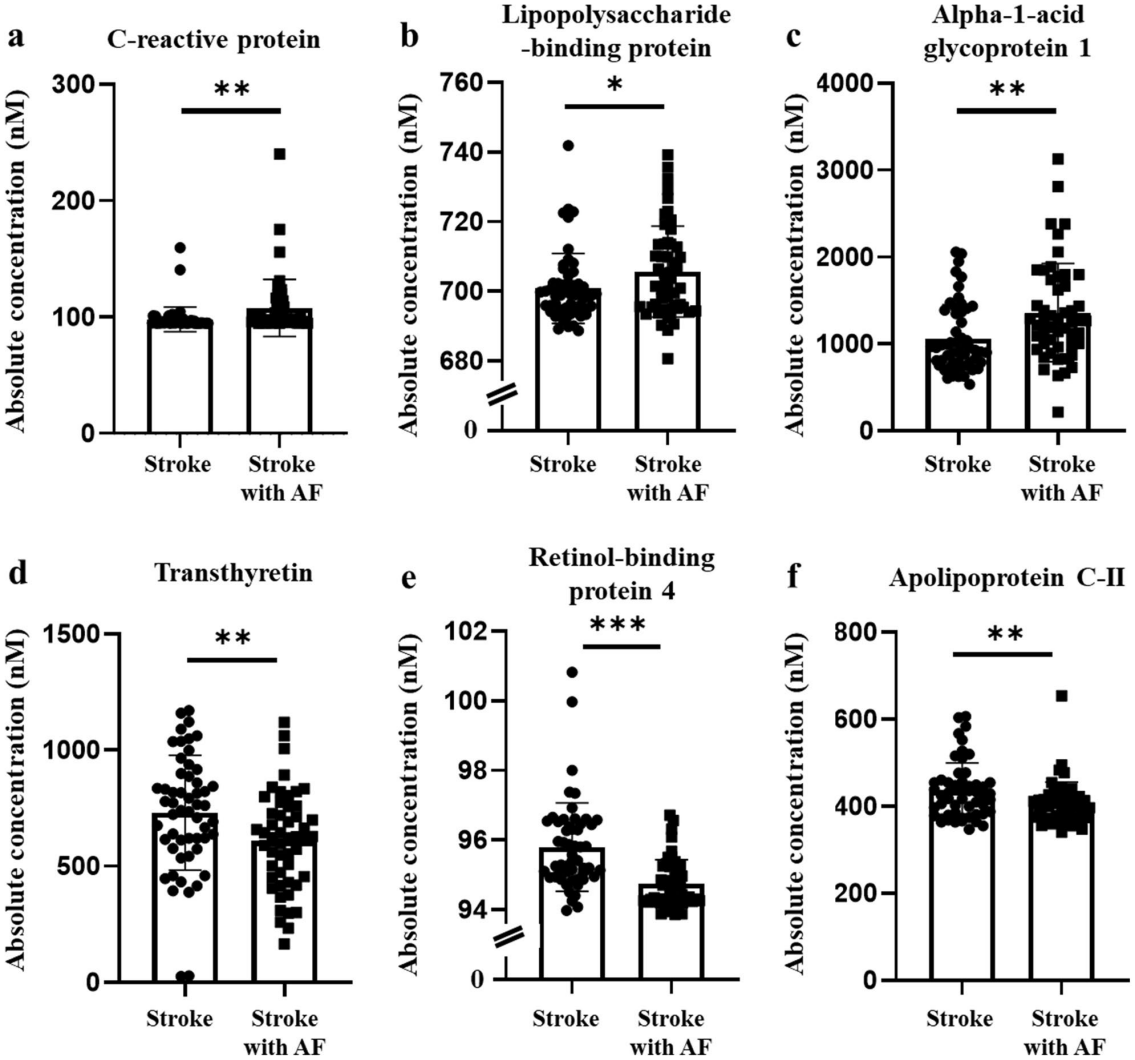
Final candidate biomarkers verified using multiple reaction monitoring. Among the 11 selected proteins, six were assessed using multiple reaction monitoring. The results revealed a significant increase in the levels of the following acute phase proteins: alpha-1-acid glycoprotein 1, C-reactive protein, and lipopolysaccharide-binding protein. Alternatively, transthyretin, apolipoprotein C-II, and retinol-binding protein 4 were decreased in patients with stroke compared to those in patients with stroke without atrial fibrillation. *p < 0.05, **p < 0.005, ***p < 0.0005.

Of the 11 selected biomarkers, 6 were assessed using MRM (Fig. 3). The results revealed a significant increase in the levels of the following acute phase proteins: alpha-1-acid glycoprotein 1 (A1AG1), C-reactive protein (CRP), and lipopolysaccharide-binding protein (LBP). In contrast, the levels of transthyretin (TTR), apolipoprotein C-II (ApoC2), and retinol-binding protein 4 (RBP4) were lower in patients with stroke and AF compared to those without AF. Protein network mapping analysis of proteins from patients with stroke and AF confirmed that retinoid metabolism and transport, G alpha (i) signaling events, retinoid cycle disease events, canonical retinoid cycle in rods (twilight vision), innate immune system, immune system, amyloid fiber formation, chylomicron assembly, chylomicron remodeling, complement cascade, platelet degranulation, and the terminal pathway of the complement system were linked to the six protein biomarkers that we analyzed (Table 3).

**Table 2:** Pathway map analysis of final candidate biomarkers.

Reactome Pathways	Observed gene (count)	Background gene (count)	FDR	TTR	APOC2	RBP4	CRP	A1AG1	LBP
Retinoid metabolism and transport	4	44	0.00026	TTR	APOC2	RBP4			
G alpha (i) signalling events	5	396	0.0098	TTR	APOC2	RBP4			
Retinoid cycle disease events	2	12	0.0135	TTR		RBP4			
The canonical retinoid cycle in rods (twilight vision)	2	23	0.0327	TTR		RBP4			
Innate Immune System	8	1025	0.0014	TTR			CRP	A1AG1	LBP
Immune System	9	1956	0.0098	TTR			CRP	A1AG1	LBP
Amyloid fiber formation	4	78	0.00076	TTR					
Chylomicron assembly	2	10	0.0116		APOC2				
Chylomicron remodeling	2	10	0.0116		APOC2				
Complement cascade	3	57	0.0093				CRP		
Platelet degranulation	3	127	0.0327					A1AG1	
Terminal pathway of complement	2	8	0.0098						

## Discussion

A1AG1, CRP, and LBP were upregulated, while TTR, ApoC2, and RBP4 were downregulated in patients with stroke and AF, compared to patients with stroke without AF. The upregulated proteins are acute/chronic inflammatory proteins. CRP plays a vital role in regulating inflammatory mechanisms. CRP performs essential functions, such as regulating the complement pathway, promoting apoptosis and phagocytosis, triggering nitric oxide release, and influencing cytokine production. LBP enhances the host response to lipopolysaccharide (LPS), a significant component of the outer membrane of gram-negative bacteria, and is activated by inflammatory mediators (e.g., cytosines) and directly or indirectly by LPS itself. A1AG1 is an acute-phase protein produced in the liver and peripheral tissues in response to systemic conditions^6,7^. Ultimately, the decreased serum concentration of TTR is due to the inflammation response^8–10^.

**Figure 4:**
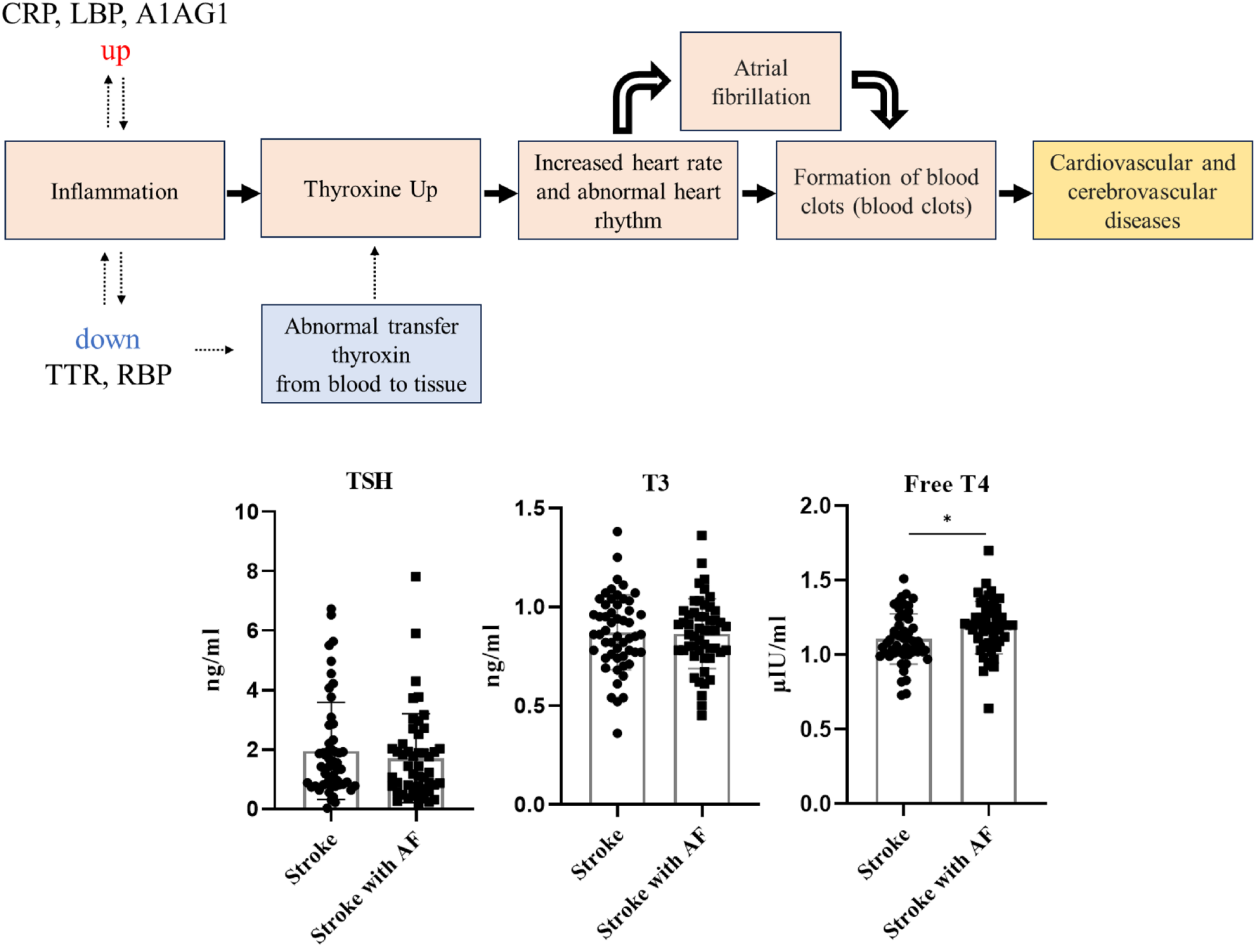
Hypothesis of activated thrombus formation in patients with stroke and atrial fibrillation. In all stroke cohorts, irrespective of AF presence, individuals with stroke exhibited elevated levels of inflammatory response-related proteins (CRP, LBP, and A1AG1) compared to those in the control group. Reduced levels of TTR and RBP were confirmed as a consequence of the inflammatory response. Notably, among patients with stroke and AF, there was a significant increase in inflammatory response-related proteins (CRP, LBP, and A1AG1) compared to patients without AF, accompanied by significant decreases in TTR and RBP. AF, atrial fibrillation; CRP, C-reactive protein; LBP, lipopolysaccharide-binding protein; A1AG1, alpha-1-acid glycoprotein 1; TTR, transthyretin; RBP, retinol-binding protein 4.

Physiological processes, such as protein production and degradation, and the immune response, are altered with increased inflammatory response, thus, causing thyroid hormone disturbances. Due to inflammation, the thyroid gland releases excessive amounts of thyroid hormones into the bloodstream, causing hyperthyroidism. The serum concentration of TTR decreases during inflammatory responses^8–10^. TTR decreases blood thyroxine (T4) levels, binding to approximately 15–20% of the thyroid hormones in the plasma^11,12^. This bond occurs because of the affinity between TTR and thyroid hormones and is involved in the transport of T4 from the blood to the brain^11,12^. Retinol-binding protein 4 (RBP4), which is decreased in patients with stroke and AF, forms a tertiary complex with TTR, which is involved in the blood transport of thyroid hormones^13^. In support of this hypothesis, we observed increased free T4 in patients with stroke and AF (Fig. 4).

Because T4 accelerates the metabolic rate, upregulated T4 increases energy consumption and promotes metabolic activities related to the cardiovascular system leading to increased cardiac activity and vasodilation, which can cause the heart to contract and relax more quickly, thereby increasing heart rate. Both hyperthyroidism and subclinical hyperthyroidism increase the risk of developing AF. In patients with stroke and AF, which causes irregular heart rhythm, elevated levels of inflammatory proteins contribute to the formation of blood clots, causing heart failure and stroke and influences the progression of these conditions. Therefore, additional treatment and management should be provided to control the inflammatory response and suppress thrombus formation in patients with stroke and AF.

Like the results of this study, a previous study reported that high free T4 levels increase the risk of AF^14^. Likewise, hyperthyroidism is reported to be a risk factor for AF^15–18^. Regarding AF as well as stroke risk, there are reports that the risk of stroke increases in the presence of thyroid disease^14,15,18^, but conflicting results showing that there is no link between thyroid disease and stroke risk have also been confirmed^19^. Moreover, it has been reported that hyperthyroidism increases the risk of thrombotic attacks in patients with AF^20^. As such, it is known that thyroid hormones are related to the risk of developing cardiovascular diseases such as stroke and AF, but this study identified protein change in stroke compared to healthy controls. At the same time, proteins changed at a higher level in stroke with AF, and it was confirmed that the changed proteins were related to an increase in thyroid hormones. Therefore, it is meaningful that the mechanism specifically enhanced in the presence of AF, in addition to changes in general stroke patients, was explained in connection with the expression of a specific proteins. In other words, through serum protein profiling in patients with stroke and AF and patients with stroke but without AF, traces of inflammatory activity such as CRP, LBP, and A1AG1 were confirmed in patients with stroke and AF, and changes in thyroid hormone transport protein. Accordingly, an increase in free T4 levels was confirmed, and a molecular mechanism was confirmed that patients with stroke and AF are more vulnerable to thrombus formation due to increased thyroid hormone levels resulting from inflammatory activity. Therefore, testing thyroid hormones, in addition to this AF-related biomarker, such as TTR, may be helpful in distinguishing stroke subtypes related to AF in patients with stroke.

Patients with AF-related stroke require anticoagulant treatment for secondary prevention purposes. However, the AF detection rate through traditional electrocardiogram and holter monitoring is not high, so additional testing (implantable cardiac monitoring) is required. Therefore, among the various subtypes of stroke, using a discovered biomarker that can predict AF-related stroke can be helpful in terms of cost-effectiveness as it can be applied to patients with stroke with a high probability of detecting AF. In addition, by routinely testing this AF-related stroke biomarker and thyroid hormone in the management of stroke patients, it will be possible to predict thrombus formation and various complications caused by AF among patients with stroke. Understanding these molecular changes can be used as evidence in establishing treatment strategies. Previous studies have shown opposing effects of hyperthyroidism and hypothyroidism on stroke recurrence, leading to speculation that thyroid dysfunction may increase the risk of recurrence in patients with AF-related stroke. Therefore, it may provide insight into developing relapse prevention treatments targeting thyroid hormone transport proteins such as TTR.

This study has some limitations. First, it is a cross-sectional study, so it is difficult to prove a causality and can only discuss correlation. Therefore, to apply the biomarkers in the clinical field, longitudinal research must be conducted. In other words, the current study collected samples at one point after admission to the hospital when the diagnosis was made. However, to prove causality, a longitudinal study with long-term follow-up of thyroid function and stroke occurrence in patients with AF without a history of stroke is needed. In addition, to predict activated thrombus formation and various complications in patients with AF-related stroke, follow-up studies should be conducted to track changes in a group or individual over a long period and investigate the efficacy of the corresponding biomarker. For example, it is necessary to confirm changes in the expression levels of thyroid hormone or AF-related stroke diagnostic biomarkers, such as TTR by obtaining samples at several time points, such as before stroke occurrence in patients with AF, at occurrence, after occurrence, and after recovery. We will be able to investigate various complications related to AF-related stroke and expand the use of biomarkers through correlation analysis. To achieve this, long-term follow-up studies using a large patient cohort are needed.

Second, the serum used in this study was obtained without anticoagulants, and coagulation occurs during the sample acquisition process. There may be limitations in detecting specific proteins, such as coagulation factors, and identifying changes in the biological environment related to them. However, because MS is a very sensitive process, introducing anticoagulants significantly impacts the performance of detecting proteins in the blood. For example, the anticoagulant used in plasma separation may act as an interfering substance during MS, affecting the overall identification of proteins in blood. That is, it interferes with the detection of proteins present in relatively low concentrations. Alternatively, serum is considered more suitable than plasma for research to discover biomarkers because it can show significant quantitative changes in certain substances due to the influence of anticoagulants^21–25^. For this reason, many studies use serum as an analysis sample in biomarker discovery research. In addition, it is important in this study to confirm the relative differences with the comparison group rather than an absolute quantitative evaluation of proteins that change in vivo. In serum separation, during the blood coagulation process, blood coagulation factors are consumed, and even if there is an absolute quantitative difference, not all substances can be consumed. Therefore, if the process for obtaining serum between samples is controlled, the relative differences between comparison groups can be sufficiently confirmed. In order to reduce variables between comparison groups as much as possible, the time from blood collection to serum separation was strictly controlled for all samples, serum separation tubes of the same manufacturer and quality were used, and the samples were kept at -70 °C until analysis immediately after separation. We tried to remove confounding variables when strictly analyzing the preprocessing process.

Despite these limitations, in this study, the serum protein profiles of patients with stroke with and without AF were analyzed to explain the thrombus formation process following stroke. In all stroke groups, regardless of the presence of AF, an increase in proteins (CRP, LBP, and A1AG1) involved in the inflammatory response was observed in patients with stroke compared to the control group. Alternatively, a decrease in TTR and RBP expression levels was observed in patients with stroke compared to the healthy controls, and this might have occurred due to the stroke-induced inflammatory response. Interestingly, in patients with stroke and AF, proteins involved in the inflammatory response (CRP, LBP, and A1AG1) were significantly increased compared to those without AF, and TTR and RBP were significantly decreased. This study proposes that patients with stroke, who commonly share an inflammation-mediated thyroid hormone alteration leading to thrombus formation, may be more vulnerable to thrombosis in the presence of AF. This study showed the mechanism of thrombus formation by the change of thyroid hormone level by linking this mechanism with the differentially expressed specific proteins in AF-related stroke. In such patients, the inflammatory response is further intensified, and the resulting abnormalities in the thyroxine transfer contribute to increased susceptibility to thrombus formation.

In conclusion, an increase in acute and chronic inflammatory proteins confirmed the activated inflammatory response observed in patients with stroke and AF. Inflammation is closely associated with cardiovascular disease and AF. Furthermore, the resulting elevation in thyroid hormone levels suggests a potential contribution to thrombus formation pathways by inducing increased cardiac activity and vasodilation. Understanding and regulating these pathological mechanisms is crucial for treating and managing patients with stroke and AF.

## Methods

### Participants

This study's methodology received approval from the Eulji University Institutional Review Board, and all participants provided informed consent. Serum protein profiling was conducted using liquid chromatography-tandem mass spectrometry (LC–MS/MS). Following this, candidate biomarkers identified in the discovery set underwent validation through multiple reaction monitoring (MRM) analyses, facilitating accurate measurement of these biomarkers.

**Table 3:** Demographic information.

Sample set		Discovery set				Validation set		
		ST with AF* (n = 48)	ST without AFʹ (n = 50)	HC^†^ (n = 99)	p-value	ST with AF* (n = 51)	ST without AFʹ (n = 54)	p-value
Sex	Male/Female	25/23	30/20	50/49	0.5377†	35/16	36/18	0.8383†
Age	Average ± SD	76.48 ± 10.39	69.46 ± 14.25	56.94 ± 4.60	0.0060¶	71.46 ± 11.45	70.92 ± 11.62	0.0030¶
BMI	Average ± SD	23.38 ± 3.64	23.68 ± 3.68	–	0.6995¶	23.95 ± 4.14	25.21 ± 3.34	0.0979¶
Thyroid function tests								
TSH	ng/ml	2.32 ± 3.37	1.50 ± 0.50	–	0.6369¶	2.22 ± 2.96	2.21 ± 2.46	0.5972
T3	ng/ml	0.84 ± 0.24	0.90 ± 0.13	–	0.1319¶	0.86 ± 0.17	0.87 ± 0.19	0.7051
Free T4	μIU/ml	1.27 ± 0.28	1.14 ± 0.22	–	0.0035¶	1.18 ± 0.17	1.11 ± 0.17	0.0234¶
Arrive time at hospital (AT)	AT < = 3 h	21	20	–	0.7072†	23	21	0.2517†
	3 < AT < = 6 h	3	3	–		5	4	
	6 < AT < = 12 h	3	2	–		5	6	
	12 < AT < = 24 h	7	5	–		2	9	
	24 < AT < = 36	4	2	–		3	5	
	36 < AT < = 48 h	4	0	–		1	1	
	AT > 48 h	3	9	–		6	8	
	AT > 7d	0	0	–		0	0	
Initial NIHSS	Average ± SD	4.58 ± 5.06	2.76 ± 2.68	–	0.1645¶	5.78 ± 6.00	3.22 ± 3.28	0.0467¶
Risk Factor	(Yes/No)							
Previous transient ischemic attacks		1/44	1/45	–	> 0.9999†	0/45	1/53	> 0.9999†
Previous stroke		13/32	11/35	–	0.6398†	33/12	12/42	< 0.0001†
Peripheral arterial disease		0/45	0/46	–	> 0.9999†	0/45	2/52	0.4991†
Coronary heart disease		12/33	4/42	–	0.0295†	5/40	6/48	> 0.9999†
Active cancer		2/43	3/43	–	> 0.9999†	1/44	6/47	0.1203†
Hypertension		36/9	37/9	–	> 0.9999†	39/6	46/8	> 0.9999†
Diabetes mellitus		11/34	13/33	–	0.8127†	18/27	28/26	0.3120†
Hyperlipidemia		5/40	18/28	–	0.0033†	17/28	18/36	0.6775†
Smoking		10/35	15/31		0.3487†	14/31	23/31	0.2984†

*ST with AF; stroke with atrial fibrillation, ʹ ST without AF; stroke without atrial fibrillation ^†^ HC; healthy controls.

Discovery set; with AF missing value 3 people, without AF missing value 4 people, validation set; with AF missing value 6 people, without AF missing value 0 people.

^¶^*p* value indicates significance between ST with AF and ST without AF using t-test. †*p* value calculated by chi-square test.

### Serum sample collection and determination of protein concentration and tryptic digestion

Serum samples collected without anticoagulants were processed for mass spectrometry (MS) analysis following established protocols from previous literature^26–36^. Blood samples were incubated for 2 h at 24 °C and centrifuged at 4000 × g for 5 min to separate the serum. A multiple-affinity removal system targeted six highly abundant proteins from the serum, leaving only low-abundance proteins. Next, a Nanosep Centrifugal Device equipped with an Omega™ Membrane 3 K was used for protein enrichment (Pall Corporation, Port Washington, NY, USA). Serum protein levels were assessed using a Bicinchoninic acid assay Protein Assay Kit (Thermo Fisher Scientific, Cleveland, OH, USA) following manufacturer instructions. Each sample contained 100 μg of protein in 50 μL. Samples were then reduced with 5 mM Tris (2-carboxyethyl) phosphine (Pierce, Rockford, IL, USA) at 37 °C, followed by centrifugation at 400 rpm for 30 min. Alkylation was performed using 15 mM iodoacetamide (Sigma-Aldrich, St. Louis, MO, USA) at 25 °C for 1 h in darkness. Enzymatic cleavage of serum proteins into peptides was achieved overnight at 37 °C using MS-grade trypsin gold (Promega, Madison, WI, USA). Afterward, the cleavage products were desalinated using a C18 cartridge from Waters (Milford, MA, USA), then the purified samples were dried with a vacuum dryer (Scan Vac, LaboGene, Lynge, Denmark). LC column injection was followed by a separate collection of eluted samples.

### Sample protein fractionation and LC–MS/MS analysis

Following the provided instructions, each sample's serum proteins were fractionated into 12 parts using a 3100 OFFGEL Low Res Kit (pH 3–10; Agilent Technologies). The resulting cleavage products underwent purification with C18 Macro spin columns (Harvard Apparatus; Holliston, MA, USA) and subsequent drying using a vacuum dryer (Scan Vac; LaboGene). These 12 samples were then subjected to protein content analysis using an Eksigent nanoLC 400 system (AB Sciex, Concord, ON, Canada) with a TripleTOF 5600 + mass spectrometer (AB Sciex, Concord, ON, Canada). Then, each sample (1 µg/µL) was injected into an Eksigent ChromXP nanoLC trap column (350 µm i.d. × 0.5 mm, ChromXP C18 3 µm) at a flow rate of 5000 nL/min. Elution occurred using an Eksigent ChromXP nanoLC column (75 µm i.d. × 15 cm) at a flow rate of 300 nL/min for 95 min. The mobile phase (B buffer) was gradually flowed into the column (ranging from 5–90%) over the 95 min, following this gradient: 0 min/mobile phase B 5%, 10.5 min/40%, 80 min/90%, and 95 min/5%.

### Synthesis and purification of label-free standard peptides

We synthesized peptides for absolute quantification using Peptron Co. (Yousung, South Korea), targeting 11 proteins identified as potential biomarkers in patients with stroke and AF. Peptides were synthesized based on specific criteria, including the absence of miscleaved sites, unmodified nature, lack of methionine, length ranging from 7–15 residues, and a low false discovery rate (< 1). Following synthesis, serial dilutions from 1 μM maximum concentration were conducted using High performance liquid chromatography-grade water, following the manufacturer's instructions.

### Label-free quantification through MRM analysis

Utilizing a skyline library, Q1/Q3 ion pairs of the selected peptides were identified. MRM parameters, including collision energy, declustering potential, and collision cell exit potential, were determined through the skyline library. Sample separation occurred using an SCIEX Exion LC system equipped with an ACQUITY UPLC BEH C18 Column (130 Å, 1.7 µm, 2.1 mm × 150 mm) and an ACQUITY UPLC BEH C18 VanGuard Pre-column (130 Å, 1.7 µm, 2.1 mm × 5 mm). Analysis was conducted with a QTRAP 5500 mass spectrometer (AB Sciex, Concord, ON, Canada). Mobile phase A contained 0.1% formic acid in HPLC-grade water. A gradient ranging from 5%–90% mobile phase B was employed during a total run time of 30 min. Mobile phase B, consisting of 0.1% formic acid in HPLC-grade acetonitrile, was introduced gradually by the LC column as follows: 1 min at 5% mobile phase B, 20 min at 40% mobile phase B, 21–25 min at 90% mobile phase B, and 25.5–30 min at 5% mobile phase B. MRM analysis for specific source parameters was as follows: curtain gas at 30 psi, low collision gas, ion spray voltage at 5500 V, temperature set at 400 °C, ion source gas 1 at 40 psi, and ion source gas 2 at 60 psi.

### Statistical analysis

The peak detection was executed utilizing the PeakView software, while total sum normalization was performed using MarkerView software. Raw data obtained from MRM analysis were processed with the MultiQuant software to ascertain absolute analyte concentration. Group comparisons were conducted using the Brown-Forsythe and Welch analysis of variance tests, followed by Dunnett's multiple comparison correction. Statistical analyses were carried out using GraphPad Prism software, version 5.0 (La Jolla, CA, USA).

### Ethics approval

The study was conducted in accordance with the Declaration of Helsinki, and approved by the Institutional Review Board of Eulji University Hospital.

### Consent to participate and publish

Informed consent was obtained from all participants involved in the study.

## Data Availability

The data presented in this study are available on request from the corresponding author.
